# Evaluation of conductivity tensor image along the perivascular space in the brains of patients with cognitive impairments

**DOI:** 10.3389/fnagi.2026.1794175

**Published:** 2026-04-28

**Authors:** Jehyeuk Ahn, Sang-Young Kim, Mun Bae Lee, Oh-In Kwon, Hak Young Rhee, Yu Jin Jung, Yoon Kim, Soonchan Park, Chang-Woo Ryu, Jinseok Lee, Geon-Ho Jahng

**Affiliations:** 1 Department of Biomedical Engineering, College of Electronics and Information, Kyung Hee University, Yongin-si, Gyeonggi-do, Republic of Korea; 2 MR Clinical Science, Health Systems, Philips Healthcare, Seoul, Republic of Korea; 3 Department of Mathematics, College of Basic Science, Konkuk University, Seoul, Republic of Korea; 4 Department of Neurology, Kyung Hee University Hospital at Gangdong, Kyung Hee University College of Medicine, Seoul, Republic of Korea; 5 Department of Radiology, Kyung Hee University Hospital at Gangdong, Kyung Hee University College of Medicine, Seoul, Republic of Korea

**Keywords:** ALPS index, Alzheimer’s disease, CTI-ALPS, DTI-ALPS, mild cognitive impairment

## Abstract

**Background:**

The diffusion tensor imaging analysis along the perivascular space (DTI-ALPS) index has been proposed as an imaging marker of impaired perivascular water transport across the Alzheimer’s disease (AD) continuum. Whether a conductivity-based ALPS derived from conductivity tensor imaging (CTI) provides a distinct physiological perspective remains to be explored. This work introduces the CTI-ALPS index. The purpose of this study was (1) to introduce the CTI-ALPS index and (2) to evaluate both the CTI-ALPS and DTI-ALPS indices in cognitively normal (CN) older adults, amnestic mild cognitive impairment (aMCI), and mild-to-moderate AD.

**Methods:**

In this prospective cross-sectional study, 110 participants (CN, *n* = 30; aMCI, *n* = 52; AD, *n* = 28) underwent diffusion MRI (*b* = 800 and 2,000 s/mm^2^) and magnetic resonance electrical property tomography (MREPT)to calculate DTI-ALPS and CTI-ALPS, respectively. Diagnostic performance and correlation with cognitive function were evaluated.

**Results:**

CTI-ALPS showed lower in AD but did not differ significantly across groups and demonstrated weaker associations with Mini-Mental State Examination (MMSE) scores. In age-adjusted ROC analyses for differentiating AD from CN, CTI-ALPS achieved modest discrimination, whereas DTI-ALPS provided slightly higher diagnostic performance.

**Conclusion:**

CTI-ALPS demonstrated a non-significant trend towards a reduction in AD and modest diagnostic utility, with weaker clinical associations than DTI-ALPS in this cohort. As an initial exploratory study, conductivity-based ALPS may serve as a distinct physical contrast reflecting ionic physiological perivascular marker, alongside diffusion-based measures, and warrants further validation with larger, age-matched datasets and reproducibility-focused designs.

## Highlights

- CTI-ALPS trended lower in Alzheimer’s disease but did not differ significantly across groups, suggesting limited dynamic range and/or greater measurement variability for conductivity-based perivascular anisotropy in this cohort.
- The right CTI ALPS was significantly negatively correlated with age and was not significantly correlated with Mini-Mental State Examination (MMSE) scores.
- CTI-ALPS and DTI-ALPS demonstrated concordant directional changes but differed in clinical sensitivity, providing perivascular information from electrical and diffusion contrasts, respectively.

## 1 Introduction

Alzheimer’s Disease (AD) is a progressive neurodegenerative disorder and the primary cause of dementia worldwide, characterized by the pathological accumulation of amyloid-beta plaques and tau tangles (Querfurth and LaFerla, 2010). These protein aggregates lead to widespread neuronal loss and brain atrophy, particularly within the hippocampus and medial temporal lobe, which serve as established imaging markers for diagnosis and staging (Jack et al., 2024). Recent research has highlighted the role of the glymphatic system—a perivascular network responsible for waste clearance and fluid balance—in the pathogenesis of AD (Iliff et al., 2012; Rasmussen et al., 2018; Tarasoff-Conway et al., 2015). Mild Cognitive Impairment (MCI), specifically the amnestic subtype (aMCI), often serves as a precursor to AD, representing a critical window where glymphatic dysfunction may first become detectable (Petersen, 2011).

To non-invasively evaluate the perivascular network responsible system in the brain, diffusion tensor imaging along the perivascular space (DTI-ALPS) has emerged as a significant neuroimaging index (Taoka et al., 2017). By measuring the directionality and magnitude of water diffusion (proton motion) along medullary veins, the ALPS index serves as a proxy for glymphatic-related fluid transport. Previous studies have demonstrated that DTI-ALPS indices are significantly lower in patients with AD compared to cognitively normal (CN) individuals, suggesting that impaired water motion correlates with increased disease severity and cognitive decline (Khalafi et al., 2026; Taoka et al., 2017).

While DTI explains proton movement, conductivity tensor imaging (CTI) focuses on the movement of ions within brain tissue. CTI is an advanced technique derived from magnetic resonance electrical property tomography (MREPT), which maps high-frequency conductivity (HFC) by solving Maxwell’s equations (Gurler and Ider, 2017). By integrating DTI information, CTI can measure the anisotropy of electrical conductivity, providing insights into how ionic mobility correlates with structural and functional changes. Because CTI-ALPS is derived from conductivity anisotropy informed by microstructural compartment modeling, it is expected to be sensitive to the extra-neurite ionic milieu (e.g., apparent ion concentration and mobility) and membrane-related hindrance that shape conductivity along perivascular pathways. Accordingly, CTI-ALPS may capture a conductivity-weighted facet of perivascular physiology that is not fully explained by water diffusion alone. Recent evidence suggests that electrical properties are altered in the AD brain; specifically, HFC and extra-neurite conductivity (EC) have been found to increase in AD patients and correlate negatively with mini-mental state examination (MMSE) scores in regions like the insula (Hong et al., 2024; Kwon et al., 2024; Park et al., 2022).

The dysregulation of ion homeostasis in AD has been studied. The failure of the brain’s electrical infrastructure—the ions that maintain membrane potential and signaling—plays a critical role in neurodegeneration. Ionic shifts in the CSF are central to the disease’s bioelectric failure. The primary driver of these shifts is the dysfunction of the Na+/K+-ATPase pump and the disruption of the blood-brain barrier (BBB) and blood-CSF barrier, which normally maintain strict ionic gradients (Lyckenvik et al., 2025). Conductivity in AD patients can be altered by ions, such as sodium (Na^+^), potassium (K^+^), Chloride (Cl^–^), Calcium (Ca^2+^), and other metal ions.

Despite the established utility of DTI-ALPS, it remains uncertain whether a conductivity-based approach could provide any information regarding perivascular health, especially in patients with cognitive impairments. Building on the principled extension of periventricular geometry to electrical properties, this study introduces the CTI-ALPS index to evaluate ion conductivity along the perivascular space. To date, no study has investigated CTI-ALPS in neurological conditions. Therefore, the objective of this study was to derive the CTI-ALPS index and compare its diagnostic performance and clinical correlations with the DTI-ALPS index across the spectrum of cognitive impairment, including CN older adults, aMCI, and mild-to-moderate AD. We hypothesized that CTI-ALPS could serve as a novel index of perivascular ion flow that is altered in AD, offering a physiologically grounded extension to traditional diffusion-based measures.

## 2 Materials and methods

### 2.1 Participants

The Institutional Review Board (IRB) approved this cross-sectional prospective study (IRB khnmc2019-07-007), and informed consent was obtained from the participants between August 2019 and December 2024. Participants provided a detailed medical history and underwent a neurologic examination, standard neuropsychological testing, and an MRI scan. Cognitive function was assessed using the full version of a Korean standardized neuropsychological test battery, known as the Seoul Neuropsychological Screening Battery (Ahn et al., 2010). Global cognitive ability was evaluated with the Korean version of the Mini-Mental State Examination (K-MMSE) and the clinical dementia rating (CDR).

Inclusion criteria were: (i) older adults meeting the study age criterion (age > 60 years); (ii) patients with mild or moderate Alzheimer’s disease (AD) diagnosed according to the National Institute of Neurological and Communicative Disorders and the Stroke–Alzheimer’s Disease and Related Disorders Association criteria (Blacker et al., 1994; Dubois et al., 2007); (iii) participants with amnestic MCI according to the Petersen criteria (Petersen et al., 2014; Petersen et al., 1999), and (iv) cognitively normal (CN) elderly participants. Exclusion criteria were: (i) severe AD; (ii) non-amnestic MCI; (iii) incomplete study participation or missing key assessments/imaging; (iv) incomplete MR images with artifacts (e.g., metal artifact); (v) other neurologic/psychiatric diseases; and (vi) brain parenchymal lesions such as severe small vessel disease, and tumor.

**FIGURE 1:**
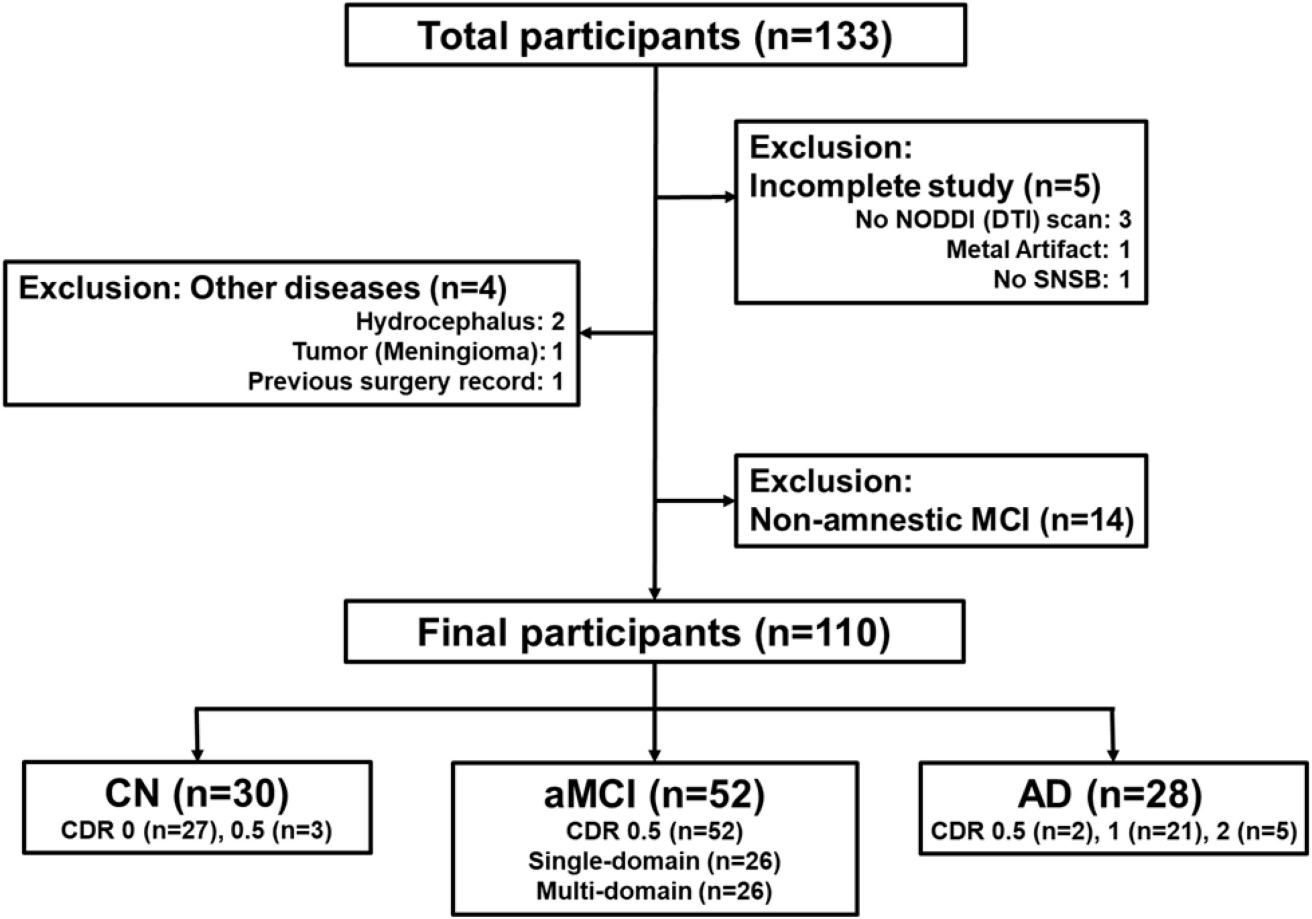
Flowchart of the summary of the selection process of participants in the study. Of the 133 initially screened participants, we excluded 36 participants due to incomplete study (*n* = 5), non-amnestic MCI (*n* = 14), and other diseases (*n* = 4). Finally, this study includes a total of 110 participants, grouped as 30 in cognitively normal (CN), 52 in amnestic mild cognitive impairment (aMCI), and 28 in Alzheimer’s disease (AD).

Figure 1 illustrates the flowchart summarizing the participant selection process in the study. Of the 133 initially screened participants, we excluded 36 participants due to incomplete study (*n* = 5), non-amnestic MCI (*n* = 14), and other diseases (*n* = 4). Finally, this study includes a total of 110 participants, grouped as 30 in CN, 52 in aMCI, and 28 in AD. Supplementary Table S1 summarizes the demographic characteristics and the neuropsychological test results of the participant groups.

### 2.2 MRI acquisition

#### 2.2.1 MREPT imaging

For the brain MREPT images, a multi-echo turbo spin-echo pulse sequence was used to calculate CTI ALPS with the following imaging parameters (Park et al., 2022): repetition time (TR) = 3,200 ms, first echo time (TE) = 12 ms with 12 ms intervals, flip angle (FA) = 90°, number of echoes (NE) = 6, number of averages (NSA) = 1, slice thickness = 5 mm, number of slices = 20 without a gap between the slices, slice orientation = transverse, and acquired voxel size = 2 × 2 × 5 mm^3^. The scan time of the MREPT sequence was 6 min and 5 s. Real and imaginary images were saved for reconstructing the HFC map.

#### 2.2.2 Diffusion tensor imaging (DTI)

For obtaining diffusion MR images to calculate DTI ALPS, a single-shot spin-echo echo-planar imaging (SS-SE-EPI) pulse sequence was used with two *b*-shells of nominally 800 and 2,000 s/mm^2^ with 16 and 32 gradient directions, respectively. In addition, *b* = 0 images were also acquired with six averages. To reduce the MR scan time for practical implementation, a relatively small number of diffusion gradient directions was adopted. The imaging parameters were as follows: TR/TE = 15,000/86 ms, FOV = 220 × 220 × 108 mm^3^, voxel size = 2 × 2 × 2 mm^3^, number of slices = 54, EPI factor = 37, and the number of excitations = 1. Total scan times were 2 min 15 s for *b* = 0 s/mm^2^, 4 min 45 s for *b* = 800 s/mm^2^, and 8 min 45 s for *b* = 2,000 s/mm^2^.

#### 2.2.3 Structural imaging

For image registration and brain tissue segmentation, a sagittal structural three-dimensional (3D) T1-weighted (T1W) image was acquired with a fast field-echo (FFE) sequence. The imaging parameters were as follows: TR = 8.1 ms, TE = 3.7 ms, FA = 8°, and voxel size = 1 × 1 × 1 mm^3^. In addition, T2-weighted turbo-spin-echo, fluid-attenuated inversion recovery (FLAIR), and gradient-echo images were also acquired to evaluate any brain abnormalities. MRI was performed using a 3.0 Tesla MRI system equipped with a 32-channel sensitivity encoding head coil (Ingenia, Philips Medical System, Best, The Netherlands).

### 2.3 MRI processing, modeling, and index calculation

An overview of the full image-processing pipeline is provided in Supplementary Figure S1.

#### 2.3.1 DTI preprocessing and ALPS index calculation

Each diffusion MRI scan (*b* = 0: 10 gradient directions, *b* = 800: 16 gradient directions, and *b* = 2,000: 32 gradient directions) was first merged, yielding a total of 58 DTI volumes. The merged DTI data were denoised by Patch2Self (Fadnavis et al., 2020), which uses the entire volume to learn a full-rank locally linear denoiser for that volume. Next, a b = 0 image was used for brain extraction to get a brain mask. By using the opposing phase-encoding direction of diffusion MRI data, the susceptibility-induced distortion was corrected using FSL *topup* (Andersson et al., 2003), and subsequently, subject motion and eddy current-induced distortion were corrected using FSL *eddy* (Andersson and Sotiropoulos, 2016). Eddy-current corrected DTI data were then registered to a 3D T1W image resampled to 1.5 mm isotropic resolution, followed by registration to the MNI 2 mm template. In addition, the gradient direction table (e.g., bvec) was also rotated to match the transformation between diffusion and MNI space. Furthermore, six elements of the diffusion tensor matrix were obtained by fitting DTI data using python library (DIPY),^1^ and the other maps, such as fractional anisotropy (FA), mean diffusivity (MD), axial and radial diffusivity (AD and RD), were generated from the eigenvalues of the tensor.

For the calculation of the DTI-ALPS index, we adopted the method for the modified ALPS (mALPS) index for automatic region-of-interest (ROIs) placement (Zhang et al., 2021). While the conventional DTI-ALPS method relies on the placement of manual regions-of-interest (ROIs) that cover the locations where medullary veins are perpendicular to the ventricle wall, the mALPS index avoids potential subjective bias caused by manual ROI placement, as well-defined ROIs from population-averaged template space can be made along the direction of medullary veins at the uppermost layer of the lateral ventricle, thereby reducing operator dependence and improving ROI reproducibility across participants. To improve robustness and minimize operator dependence, we placed the 5-mm diameter ROIs on the bilateral projection fibers and association fibers on the color-coded FA map in the MNI space. The coordinates of the centers of the left and right ROIs were e (24, −12, 24) and (−28, −12, 24) in projection fibers, respectively, and (36, −12, 24) and (−40, −12, 24) in association fibers, respectively. We measured the mean diffusivities along the *x*-axis (Dx), *y*-axis (Dy) and *z*-axis (Dz) in the ROIs on projection (Dxproj, Dyproj, Dzproj) and association fibers (Dxassoc, Dyassoc, Dzassoc). Finally, mALPS index was calculated with the following equation: [(Dxproj + Dxassoc)/(Dyproj + Dzassoc)] (Taoka et al., 2017).

#### 2.3.2 HFC mapping from MREPT data

To reconstruct conductivity, the acquired phase information is processed to remove potential artifacts and any contributions not related to the tissue’s electrical properties, such as those resulting from magnetic field inhomogeneities. After that, the spatial distribution of the RF field (B1) is calculated from the phase images. The electric (E) field induced by the MRI RF pulse can be calculated from the B1 field using Maxwell’s equations. Once the E-field is known, the local electrical properties (conductivity and permittivity) are reconstructed by solving a partial differential equation known as the Helmholtz equation, which relates the E-field to the conductivity and permittivity (Voigt et al., 2011; Zhang et al., 2014). Therefore, the relationship between the B1 field denoted as **B_1_** and the electrical properties is expressed as:


$$\nabla^{2}{\text{B}}_{1}=i\,\omega \,\mu_{0}\,\tau_{H}\,{\text{B}}_{1}-\frac{\nabla \tau_{H}}{\tau_{H}}\times \left(\nabla \times {\text{B}}_{1}\right)$$ (1)

where ω is the angular frequency, μ_0_ = 4π × 10^−7^*N*/*A*^2^ is the magnetic permeability of the free space, and τ_*H*_ = σ_*H*_ + *i*ωϵ_*H*_ at high-frequency conductivity σ_*H*_ and permittivity ϵ_*H*_ (Katscher and van den Berg, 2017; Katscher et al., 2009; Voigt et al., 2011). The transverse field of **B_1_** can be decomposed into the positively rotating field $B_{1}^{+}=\frac{1}{2}\,\left(B_{x}+i\,B_{y}\right)$ and the negatively rotating field $B_{1}^{-}=\frac{1}{2}\,\left(B_{x}-i\,B_{y}\right).$ With the conventional MRI scanner having a single transmit channel, the magnetic field $B_{1}^{+}$ component is available. We denoted ϕ^+^ and ϕ^−^ as the phase terms of $B_{1}^{+}$ and $B_{1}^{-}$, respectively. By assuming σ_*H*_ >> ϵ_*H*_, a phase-based MREPT formula was derived as:


$$\left(\nabla \phi^{t\,r}\cdot \nabla \left(\frac{1}{\sigma_{H}}\right)\right)+\frac{\nabla^{2}\phi^{t\,r}}{\sigma_{H}}-2\,\omega \,\mu_{0}=0$$ (2)

where ϕ^*tr*^ = ϕ^+^ + ϕ^−^. To stabilize the formula (2), the MREPT formula based on a convection reaction equation can be derived by adding the regularization coefficient *c* (Gurler and Ider, 2017):


$$-c\,\nabla^{2}\left(\frac{1}{\sigma_{H}}\right)+\left(\nabla \phi^{t\,r}\cdot \nabla \left(\frac{1}{\sigma_{H}}\right)\right)+\frac{\nabla^{2}\phi^{t\,r}}{\sigma_{H}}=2\,\omega \,\mu_{0}$$ (3)

MREPT depends upon the relatively weak phase signal by a secondary RF magnetic field from the induced electrical current by the time-varying RF field. Due to the weak phase signal and noise artifacts, a multi-echo spin-echo MR pulse sequence is advantageous to reduce the noise artifacts using the weight for *k*th echo:


$$\phi^{t\,r}=\overset{N_{E}}{\underset{k=1}{\sum }}w_{k}\,\phi^{k},w_{k}=\frac{{\left|\rho_{k}\right|}^{2}}{\sum_{j=1}^{N_{E}}{\rho_{j}}^{2}}$$

where ϕ*^k^* [mymathe] and ρ_*k*_ are the phase signal and complex MR signal, respectively, for the *k-*th echo. To solve the convection reaction partial differential equation in (3), we used the 2-dimensional finite-difference method. For each image matrix, the Equation (3) is written as:


$$\left[\begin{array}{c} \vdots \\ -c\,\left(\frac{\partial^{2}}{\partial x^{2}}+\frac{\partial^{2}}{\partial y^{2}}\right)+\frac{\partial \phi^{t\,r}}{\partial x}\,\frac{\partial }{\partial x}+\frac{\partial \phi^{t\,r}}{\partial y}\,\frac{\partial }{\partial y}+\frac{\partial^{2}\phi^{t\,r}}{\partial x^{2}}+\frac{\partial^{2}\phi^{t\,r}}{\partial y^{2}}\\ \vdots \end{array}\right]$$ (4)

$$\left[\begin{array}{c} \vdots \\ \frac{1}{\sigma_{H}}\\ \vdots \end{array}\right]=\left[\begin{array}{c} \vdots \;\\ 2\,\omega \,\mu_{0}\\ \;\vdots \end{array}\right]$$

The finite-difference method for solving the Equation (4) is to find the solutions of a linear matrix system **Ax** = **b** with the appropriate processing of the Dirichlet boundary conditions. **A** is a staff matrix, $x=\left(\frac{1}{\sigma_{H\,1}},\frac{1}{\sigma_{H\,2}},\ldots ,\frac{1}{\sigma_{H\,N}}\right)$, and **b** = (2ωμ_0_, 2ωμ_0_,…, 2ωμ_0_), respectively. We used the finite-difference method to solve the above matrix system with regularization coefficient *c* = 0.03 in Equation (4) (Park et al., 2022). There are several computational algorithms developed to perform this inversion from the E-field to the desired electrical properties.

#### 2.3.3 Conductivity tensor imaging (CTI) mapping

The multi-compartment spherical mean (MC-SMT) technique has been developed to evaluate microscopic features of the intra- (restricted) and extra-neurite (hindered) compartments in the neuronal tissue, which are two different compartments of a neuron (Kaden et al., 2016). The intra-neurite compartment contains axons and dendrites and can be modeled as a collection of infinitely thin “sticks.” The extra-neurite compartment is everything else in the brain, except for neurites and free water, and can reflect the interactions of water molecules with the macromolecules, fibers, and membranes in the brain tissue. The gray matter in the brain contains the cell bodies of neurons and dendrites and axons, and the white matter in the brain contains myelinated axons.

After calculating HFC from the B1 phase, the recovered HFC σ_*H*_ was decomposed into the intra-neurite and extra-neurite compartments to calculate the compartmental conductivities (Jahng et al., 2021).


$$\sigma_{H}=\sigma_{i\,n\,t}+\sigma_{e\,x\,t}=\nu_{i\,n\,t}\,{\bar{c}}^{i\,n\,t}\,D^{i\,n\,t}+\left(1-\nu_{i\,n\,t}\right)\,{\bar{c}}^{e\,x\,t}\,D^{e\,x\,t}$$ (5)

where σ_*int*_ and σ_*ext*_ are the intra-neurite conductivity (IC) and the extra-neurite conductivity (EC), respectively, ν_*int*_ is the intra-neurite volume fraction (IVF), ${\bar{c}}^{i\,n\,t}$is the intra-neurite ion concentration, and ${\bar{c}}^{e\,x\,t}$ is the extra-neurite ion concentration (EIC). Similarly, *D^int^* and *D^ext^* are the intra-neurite diffusivity (ID) and the extra-neurite diffusivity (ED), respectively. To obtain ν_*int*_, *D^int^* and *D^ext^* in Equation (5), the MC-SMT was applied (Kaden et al., 2016). The key insight of MC-SMT is that for a specified diffusion weighting factor *b*, the spherical mean of the diffusion signal ${\bar{e}}_{b}$ over the gradient directions is invariant to the fiber orientation distribution.

Since the internal current flow at low-frequency (< 1 kHz) is only restricted to the extra-neurite space between the cells, the low-frequency dominant average scalar conductivity, *σ_*L*_*, can be expressed as


$$\sigma_{L}=\left(1-\nu_{i\,n\,t}\right)\,{\bar{c}}^{e\,x\,t}\,D^{e\,x\,t}=\frac{\left(1-v_{i\,n\,t}\right)\,D^{e\,x\,t}\,\sigma_{H}}{v_{i\,n\,t}\,\beta \,D^{i\,n\,t}+\left(1-v_{i\,n\,t}\right)\,D^{e\,x\,t}}$$ (6)

The low-frequency conductivity σ_*L*_ in Equation (6) depends on the intra-neurite volume fraction (ν_*int*_), the apparent extra-neurite ion concentration (${\bar{c}}^{e\,x\,t}$), and the extra-neurite mean diffusivity (*D^ext^*). Since CSF is a highly conductive liquid without cell membranes, the low-frequency conductivity σ_*L*_ is almost identical to the high-frequency conductivity σ_*H*_ in CSF region (Baumann et al., 1997).

For measuring the anisotropy of conductivity, we assume that the extra-neurite diffusion tensor and the water diffusion tensor share the eigenvectors. The eigenvalues, $d_{1}^{e\,x\,t}=d_{2}^{e\,x\,t}\leq d_{3}^{e\,x\,t}$, satisfy the following relations:


$$\frac{d_{1}^{e\,x\,t}+d_{2}^{e\,x\,t}+d_{3}^{e\,x\,t}}{3}=D^{e\,x\,t},d_{1}^{e\,x\,t}=d_{2}^{e\,x\,t}=\left(1-\nu_{i\,n\,t}\right)\,D^{i\,n\,t}$$ (7)

The low-frequency dominant conductivity tensor *C_L_* can be expressed as the following


$$C_{L}=\frac{\left(1-v_{i\,n\,t}\right)\,\sigma_{H}}{v_{i\,n\,t}\,\beta \,D^{i\,n\,t}+\left(1-v_{i\,n\,t}\right)\,D^{e\,x\,t}}\,S_{D}\,\left(\begin{array}{ccc} d_{1}^{e\,x\,t} & 0 & 0\\ 0 & d_{2}^{e\,x\,t} & 0\\ 0 & 0 & d_{3}^{e\,x\,t} \end{array}\right)\,S_{D}^{T}$$ (8)

where the column vectors of *S_D_* are the orthogonal eigenvectors of the water diffusion tensor and the superscript *T* denotes the transpose. Therefore, *C_L_* is also a tensor where the ion mobility is assumed to be proportional to the water molecule diffusion flow (Jahng et al., 2021; Lee et al., 2020; Marino et al., 2021; Sajib et al., 2018). CTI is an advanced tool that enhances our understanding of brain physiology and pathology by focusing on the electrical properties of brain tissues.

#### 2.3.4 CTI-ALPS mapping

To quantify perivascular anisotropy from *C_L_*, we adapt the DTI-ALPS index by replacing tensor components *D*⋯ with conductivity components *C*_*L*,⋯_. Using two white-matter ROIs—a projection-fiber ROI (superscript *p*) and an association-fiber ROI (superscript *a*)—the CTI-ALPS index is defined as


$$A\,L\,P\,S_{C\,T\,I}=\frac{C_{L,x\,x}^{\left(p\right)}+C_{L,x\,x}^{\left(a\right)}}{C_{L,y\,y}^{\left(p\right)}+C_{L,z\,z}^{\left(a\right)}}$$ (9)

With *C_L_* from Equation (8), this can be written in a coordinate-free form using direction cosines (rows of *S_D_*):


$$A\,L\,P\,S_{C\,T\,I}=\frac{\sum_{i=1}^{3}{\left(S_{x\,i}^{\left(p\right)}\right)}^{2}\,d_{i,p}^{e\,x\,t}+\sum_{i=1}^{3}{\left(S_{x\,i}^{\left(a\right)}\right)}^{2}\,d_{i,a}^{e\,x\,t}}{\sum_{i=1}^{3}{\left(S_{y\,i}^{\left(p\right)}\right)}^{2}\,d_{i,p}^{e\,x\,t}+\sum_{i=1}^{3}{\left(S_{z\,i}^{\left(a\right)}\right)}^{2}\,d_{i,a}^{e\,x\,t}}$$ (10)

With *C_L_* from Equation (8), this can be written in a coordinate-free form using direction cosines (rows of *SD*):In practice, the ALPS protocol places ROIs so that principal fiber directions align with the scanner axes (projection: *x*-dominant; association: *z*-dominant). Under this standard axis-alignment (*SD* = *I* for each ROI), Equation (7) gives Projection fiber (p),


$$d_{1,p}^{e\,x\,t}=d_{2,p}^{e\,x\,t}=\left(1-\nu_{i\,p}\right)\,D_{p}^{i\,n\,t}=\left(1-\nu_{i\,p}\right)\,D_{i\,p}$$

And, Association fiber (a),


$$d_{3,a}^{e\,x\,t}=3\,D_{a}^{e\,x\,t}-2\,\left(1-\nu_{i\,p}\right)\,D_{a}^{i\,n\,t}=3\,D_{e\,a}-2\,\left(1-\nu_{i\,a}\right)\,D_{i\,a}$$

so that the CTI-ALPS index simplifies to the closed-form


$$A\,L\,P\,S_{C\,T\,I}=\frac{\left(1-\nu_{i\,a}\right)\,D_{i\,a}+\left(1-\nu_{i\,p}\right)\,D_{i\,p}}{\left(1-{\text{íí}}_{i\,p}\right)D_{i\,p}+\left(3D_{e\,a}-2\left(1-\nu_{i\,a}\right)D_{i\,a}\right]}$$ (11)

where the subscripts *p* and *a* denote the projection and association ROIs, respectively ($\nu_{i\,p}\,\nu_{i\,n\,t}^{\left(p\right)}$, $D_{i\,p}\,D_{i\,n\,t}^{\left(p\right)}$, $D_{e\,p}\,D_{e\,x\,t}^{\left(p\right)}$; $\nu_{i\,a}\,\nu_{i\,n\,t}^{\left(a\right)}$, $D_{i\,a}\,D_{i\,n\,t}^{\left(a\right)}$, $D_{e\,a}\,D_{e\,x\,t}^{\left(a\right)}$). The *ALPS*_*CTI*_ depends solely on the extra-neurite eigenvalues via Equation (7), i.e., on ν_*int*_, *D*_*int*_, *D*_*ext*_ within each ROI. If the scalar pre-factor differs between ROIs (e.g., due to ROI-specific ion environments), the cancelation is not exact and should be modeled explicitly. In practice, ν_*int*_, *D*_*int*_, *D*_*ext*_ are estimated for each ROI using MC-SMT and mapped as ν_*ip*_, *D*_*ip*_, *D*_*ep*_ for the projection ROI and ν_*ia*_, *D*_*ia*_, *D*_*ea*_ for the association ROI. The CTI–ALPS index is obtained from Equation (11). if the ROIs are not perfectly axis-aligned, use Equation (10) with direction cosines *S_D_* from the diffusion eigenvectors; β still cancels under a common prefactor across ROIs.

### 2.4 Statistical analysis

In this study, CTI-ALPS and DTI-ALPS with *b* = 800 and *b* = 2,000 were measured from MRI data. The statistical analysis was performed using the Medcalc (MedCalc Software, Acacialaan, Ostend, Belgium), R (version 4.3.2) statistical programs, and Python (version 3.8.20) with NumPy (1.24.3), SciPy (1.10.1), pandas (2.0.3), and Matplotlib (3.7.2). Because age significantly differed between the AD and other groups, age was included as a control factor in all subsequent analyses of MRI measures.

#### 2.4.1 Group comparison

ANCOVA analyses were performed for MRI measures of CTI-ALPS and DTI-ALPS among the three participant groups with age as a covariate. If any significant differences were found, the *post-hoc* test was performed using Bonferroni correction with *p* = 0.05. Furthermore, to evaluate the laterality of the ALPS index between the right and left sides, we performed the following analyses. First, the ALPS values between the left and right sides within each participant group were compared using a paired *t*-test. Second, we calculated within-subject laterality as the difference between right and left sides of the ALPS index for each participant (Δ = RT−LT) and compared Δ among the three participant groups using ANCOVA with age as a covariate.

#### 2.4.2 Correlation analysis

First, Pearson correlation analysis was performed to evaluate the correlation between each MRI measure and age. Second, partial correlation analysis was performed to evaluate the correlation between each ALPS and MMSE scores with age as a covariate. Finally, we evaluated the correlation between MRI measures.

#### 2.4.3 Receiver operating characteristic (ROC) curve analysis

To investigate which MRI index was best to differentiate AD from others, ROC curve analysis was performed while adjusting for age. The optimal cut-off point, maximizing the combined measure of sensitivity and specificity, was determined using the Youden Index. We report sensitivity (SE), specificity (SP), and area under the curve (AUC) with *p*-value.

## 3 Results

### 3.1 Demographics

Demographic characteristics and neuropsychological test results across the CN, MCI, and AD groups are summarized in Supplementary Table S1. Age differed significantly among groups (ANOVA: *F* = 6.444, *p* = 0.002), with *post-hoc* testing showing that the AD group was older than both the CN (*p* = 0.008) and MCI groups (*p* = 0.026). Sex was not significantly different among the groups (χ^2^ ≤ 2.239, *p* ≥ 0.135). As expected, MMSE scores were significantly lower in AD than CN (*p* < 0.0001) and MCI (*p* < 0.0001).

### 3.2 Group comparison of MRI measures

Table 1 presents the ANCOVA results comparing MRI measures among the three participant groups. The CTI ALPS index did not differ significantly between the participant groups. The DTI ALPS index differed significantly between the participant groups, particularly between the CN and AD groups (Supplementary Figure S2).

**TABLE 1:** Results of comparison of MRI measures among the three participant groups.

ROIs	Side	CN (1)	MCI (2)	AD (3)	*Statistics
CTI-ALPS indices					
ALPS index	LT	1.26 ± 0.12	1.21 ± 0.14	1.18 ± 0.17	*F* = 1.162, *P* = 0.317
	RT	1.02 ± 0.11	1.03 ± 0.10	0.97 ± 0.09	*F* = 1.899, *P* = 0.155
	^#^Paired *T*-test	*P* = 0.676	*P* = 0.200	*P* = 0.186	N/A
DTI-ALPS indices					
Using *b* = 800	LT	1.41 ± 0.23	1.31 ± 0.17	1.23 ± 0.13	***F = 3.861, P = 0.024*** ***(1, 3) p = 0.0458***
	RT	1.43 ± 0.22	1.33 ± 0.20	1.24 ± 0.17	F = 3.062, *P* = 0.051
	^#^Paired *T*-test	*P* = 0.739	*P* = 0.347	*P* = 0.215	N/A
Using *b* = 2,000	LT	1.33 ± 0.18	1.27 ± 0.14	1.19 ± 0.11	***F = 3.387, P = 0.037*** ***(1, 3) p = 0.0411***
	RT	1.35 ± 0.17	1.30 ± 0.16	1.19 ± 0.14	***F = 3.183, P = 0.045*** ***(1, 3) p = 0.0430***
	^#^Paired *t*-test	*P* = 0.339	*P* = 0.358	*P* = 0.141	N/A

The continuous data are listed as mean ± standard deviation. *All data were evaluated by ANCOVA (F, *p*-value) with age as a covariate. If any significant, then the *post-hoc* test was performed using Bonferroni correction with *p* = 0.05. ^#^Paired *t*-test of the ALPS values between left and right sides for each group. DTI, Diffusion Tensor Imaging; ALPS, along the perivascular space; CTI, Conductivity Tensor Imaging; CN, cognitively normal; MCI, amnestic mild cognitive impairment; AD, Alzheimer’s disease; LT, Left; RT, Right. Italic and bold characters show a statistically significant comparison.

The results of the laterality analyses are listed in Table 1 and Supplementary Table S2. There is no significant difference ALPS index between left and right in each group (Table 1). In addition, the difference between the right and left sides of the ALPS index for each participant (Δ = RT−LT) showed no significant group differences for both CTI and DTI (Supplementary Table S2).

### 3.3 Correlation analyses of MRI measures

#### 3.3.1 With age

**FIGURE 2:**
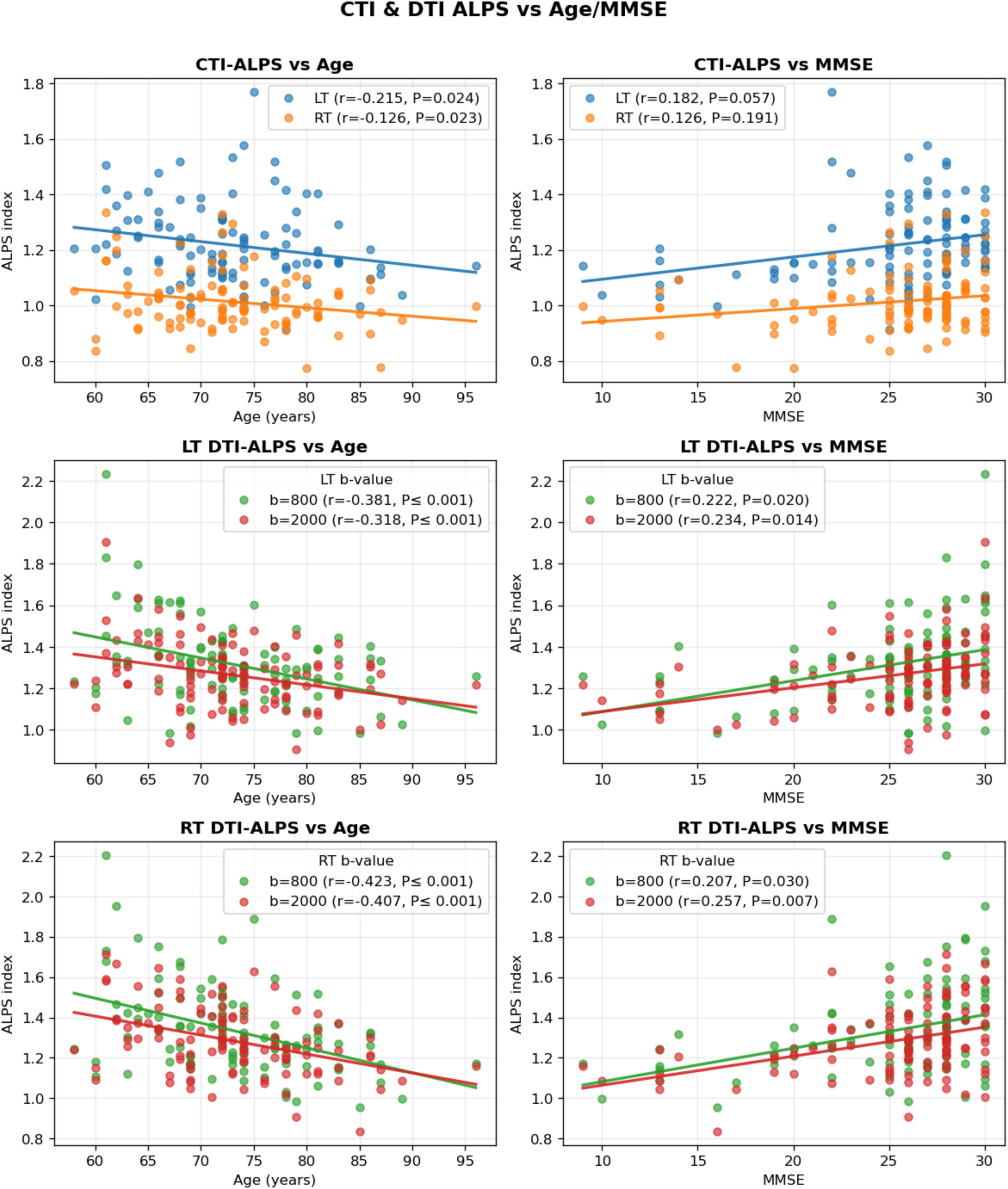
Correlations of CTI-ALPS and DTI-ALPS indices with age and cognition. Scatterplots show relationships between ALPS indices and age (left column) or MMSE (right column). Top row: CTI-ALPS versus age and MMSE for LT and RT. Middle and bottom rows: DTI-ALPS versus age and MMSE for LT and RT, shown separately for b = 800 and *b* = 2,000 s/mm^2^. Solid lines indicate linear fits. Correlation coefficients (*r*) and *p*-values are displayed within each panel. CTI-ALPS, Conductivity tensor imaging–based ALPS index; DTI-ALPS, Diffusion tensor imaging–based ALPS index; LT/RT, Left/Right; MMSE, Mini-Mental State Examination.

Table 2 lists the results of the correlation analysis between ALPS measures and age or MMSE scores. In addition, those correlation results are also shown in Figure 2. The right CTI ALPS was significantly negatively correlated with age (Table 2 and Figure 2). The DTI ALPS was significantly negatively correlated with age (Table 2 and Figure 2). These correlations indicate that conductivity-derived measures may be sensitive to normal aging-related shifts in extracellular water/ionic milieu, which can partially overlap with disease-related effects.

**TABLE 2:** Result of correlation analysis between ALPS measures and age or MMSE scores.

Index	Side	Details	Age (*r*, *p*)*	MMSE (*r*, *p*)^†^
CTI-ALPS indices				
ALPS indexes	LT	All	***r = −0.215, P = 0.024***	*r* = 0.182, *P* = 0.057
	RT	All	***r = −0.126, P = 0.023***	*r* = 0.126, *P* = 0.191
DTI-ALPS indices				
ALPS indexes	LT	*b* = 800	***r = −0.381, P ≤ 0.001***	***r = 0.222, P = 0.020***
		*b* = 2,000	***r = −0.318, P ≤ 0.001***	***r = 0.234, P = 0.014***
	RT	*b* = 800	***r = −0.423, P ≤ 0.001***	***r = 0.207, P = 0.030***
		*b* = 2,000	***r = −0.406, P ≤ 0.001***	***r = 0.257, P = 0.007***

*Pearson’s correlation coefficient r with *P*-value.

† Partial correlation coefficient r with adjusting age with *P*-value. Italic and bold characters show a statistically significant comparison. DTI, Diffusion Tensor Imaging; ALPS, along the perivascular space; CTI, Conductivity Tensor Imaging; MMSE, Mini-Mental State Examination scores; LT, Left; RT, Right.

#### 3.3.2 With MMSE scores

The CTI ALPS was not significantly correlated with MMSE scores (Table 2 and Figure 2). The DTI ALPS were positively correlated with MMSE scores (Table 2 and Figure 2).

#### 3.3.3 Between CTI-ALPS and DTI-ALPS

Lists the results of the correlation analysis between CTI-ALPS and DTI-ALPS. The CTI-ALPS was positively correlated with the DTI-ALPS (Supplementary Table S3 and Supplementary Figure S3).

#### 3.3.4 Between tensor components of CTI-ALPS and DTI-ALPS

Supplementary Table S4 lists the results of the correlation analysis between tensor components of CTI-ALPS and DTI-ALPS (Supplementary Figure S4). For both projection and association fibers, the positive correlation was found between Dxx and Cxx, between Dyy and Cyy, and between Dzz and Czz.

### 3.4 ROC analyses of MRI measures

Table 3 lists the results of the ROC curve analysis of CTI ALPS and DTI ALPS. Supplementary Figure S5 summarizes the results of the ROC curve analysis of CTI-ALPS and DTI-ALPS.

**TABLE 3:** Results of a receiver operating characteristic (ROC) curve analysis of CTI ALPS and DTI ALPS indices.

ROIs	Side	DTI b-value	CN vs. MCI				CN vs. AD				MCI vs. AD			
			SE	SP	AUC	*p*	SE	SP	AUC	*p*	SE	SP	AUC	*p*
CTI-ALPS index														
CTI-ALPS index	LT		***56***	***70***	***0.63***	***0.05***	***75***	***80***	***0.74***	***0.002***	75	63	0.62	0.082
	RT		62	53	0.51	0.942	71	57	0.64	0.077	***43***	***85***	***0.64***	***0.036***
DTI-ALPS index														
DTI-ALPS index	LT	*b* = 800	***71***	***60***	***0.65***	***0.023***	***89***	***60***	***0.78***	*** ≤ 0.001***	***75***	***54***	***0.64***	***0.036***
		*b* = 2,000	87	37	0.6	0.145	***75***	***73***	***0.75***	*** ≤ 0.001***	***79***	***58***	***0.69***	***0.007***
	RT	*b* = 800	***65***	***70***	***0.65***	***0.021***	***86***	***70***	***0.78***	*** ≤ 0.001***	***68***	***60***	***0.65***	***0.033***
		*b* = 2,000	69	47	0.58	0.234	***82***	***63***	***0.76***	*** ≤ 0.001***	***82***	***62***	***0.71***	***0.002***

DTI, Diffusion Tensor Imaging; ALPS, along the perivascular space; CTI, Conductivity Tensor Imaging; CN, cognitively normal; MCI, mild cognitive impairment; AD, Alzheimer’s disease; SE, Sensitivity; SP, Specificity; AUC, Area under the ROC curve. Italic and bold characters show a statistically significant comparison.

#### 3.4.1 Differentiation of MCI from CN

To differentiate MCI from CN, the AUC values were lower for both ALPS measures compared to the AUC values with other groups’ differentiation. Both CTI-ALPS and DTI-ALPS can differentiate between the two groups, but the sensitivity and specificity were low.

#### 3.4.2 Differentiation of AD from CN

The CTI-ALPS on the left side can differentiate between the two groups (SE = 75%, SP = 80%, AUC = 0.74, *p* = 0.002). The DTI-ALPS on the left side can differentiate between the two groups (SE = 89%, SP = 60–70%, AUC = 0.78, *p* < 0.001 in the *b*-value = 800; SE = 75%, SP = 73%, AUC = 0.75, *p* < 0.001 in the *b*-value = 2,000). The differentiation performance of DTI-ALPS on the right side was similar to that on the left (Table 3).

#### 3.4.3 Differentiation of AD from MCI

The CTI-ALPS can differentiate between the two groups (SE = 43%, SP = 85%, AUC = 0.64, *p* = 0.036). The DTI-ALPS on the left side can differentiate between the two groups (SE = 75%, SP = 54%, AUC = 0.64, *p* < 0.036 in the b-value = 800; SE = 79%, SP = 58%, AUC = 0.69, *p* < 0.007 in the *b*-value = 2,000). The differentiation performance of DTI-ALPS on the right side was similar to that on the left (Table 3).

## 4 Discussion

### 4.1 Comparative sensitivity of CTI-ALPS and DTI-ALPS

In this study, CTI-ALPS values exhibited a downward trend in AD compared to CN and MCI groups, but these differences did not reach statistical significance (Table 1). In contrast, the DTI-ALPS index demonstrated robust group-level discrimination, particularly between CN and AD cohorts. Correlation analyses further underscored this divergence: while DTI-ALPS was significantly associated with both age and MMSE scores, CTI-ALPS showed only a weak negative correlation with age and no significant association with cognitive performance (Table 2 and Figure 2). These results suggest that CTI-ALPS captures a less sensitive aspect of perivascular microstructural integrity than DTI-ALPS in this specific cohort. A primary factor may be the limited dynamic range of the CTI-ALPS metric. Because CTI-ALPS is mathematically derived from a combination of diffusion eigenvalues and volume fractions across intra- and extra-neurite compartments (Equation (11)), the specific compartmental shifts in AD may not manifest with the same magnitude as the global directional water-diffusion alterations that drive DTI-ALPS. Moreover, CTI-ALPS is a ratio across two ROIs that can partially cancel, weakening global between-group shifts. However, ROI-specific ion microenvironments or reconstruction models can increase inter-individual variance and further reduce statistical sensitivity. Previous studies have shown that a lower DTI-ALPS is associated with a higher amyloid burden and faster amyloid accumulation/progression on PET, as well as worse metabolic/neurodegenerative profiles (Kamagata et al., 2022). Such variance may be further accentuated in older cohorts, where aging-related conductivity changes could compress the effective dynamic range of CTI-ALPS and reduce sensitivity to disease-specific contrasts (He et al., 2024).

Conceptually, DTI-ALPS primarily reflects the physical movement of water molecules along the perivascular space, a proxy for glymphatic clearance, which is critical for removing neurotoxic proteins such as amyloid-beta (Iliff et al., 2012; Tarasoff-Conway et al., 2015). CTI-ALPS, however, is theoretically shaped by ionic mobility and the extracellular microenvironment. CTI-ALPS can be interpreted as reflecting perivascular ionic homeostasis and extracellular microstructural conditions (e.g. gliosis-related extracellular changes or altered membrane integrity) that may evolve independently from bulk diffusion changes. Furthermore, previous studies have reported increased high-frequency conductivity (HFC) and extra-neurite conductivity (EC) in the insula of AD patients, potentially reflecting gliosis-related changes in the extracellular space or altered membrane integrity. Although CTI-ALPS did not differentiate groups as effectively as DTI-ALPS (Table 2 and Figure 2), the two indices were positively correlated (Supplementary Tables S3, S4). Rather than a standalone diagnostic tool, CTI-ALPS serves as an exploratory marker anchored to ionic physiology, providing a different perspective on the pathological state of the extracellular environment and may warrant investigation alongside DTI-ALPS in future studies to determine its precise added value. Furthermore, beyond cross-sectional diagnostic discriminant, CTI-ALPS may have the potential to provide more useful information as a response biomarker in settings targeting ion regulation or barrier-related physiology (e.g., interventions affecting ion transporters/channels). This is because conductance changes may occur before global structural remodeling is detected by diffusion measurements. This possibility remains at the hypothesis stage in this study and needs to be evaluated together with independent biomarkers in future longitudinal or interventional studies.

The relative stability of CTI-ALPS observed in this study, in contrast to the significant decline in DTI-ALPS, may be explained by the complex ionic shift characteristic of AD pathology. While CSF sodium levels are elevated due to Na+/K+-ATPase failure, levels of other key electrolytes, such as potassium, chloride, and calcium, are typically decreased in the CSF of AD patients. Sodium and potassium are the most important ions in AD brains to govern the Na^+^/K^+^-ATPase pump. Sodium levels in the CSF of AD patients are markedly elevated, but CSF potassium levels of AD patients are significantly decreased (Mizuno et al., 2025; Vitvitsky et al., 2012). In AD, this pump’s activity is significantly depressed. Chloride (Cl^–^) homeostasis is essential for the inhibitory action of the neurotransmitter GABA. In symptomatic AD patients, CSF chloride concentrations show a significant decrease (averaging a ∼3–5% drop compared to healthy controls) (Porcher et al., 2025). The calcium (Ca^2+^) hypothesis of AD suggests that Ca^2+^ signaling disruption is a primary driver of the disease. AD neurons suffer from chronic calcium overload. Aβ can form non-specific ion pores in cell membranes, allowing unregulated Ca2+ influx (Arispe et al., 1993). While intracellular calcium is dangerously high in AD (calcium overload), CSF calcium levels are typically lower or show an age-related decline that is accelerated in AD patients (Subhash et al., 1991). Other metal ions are zinc (Zn^2+^), copper (Cu^2+^), and magnesium (Mg^2+^) (Bhoi et al., 2025). Since MREPT measures the aggregate conductivity of the tissue microenvironment, these opposing ionic shifts—hypernatremia counterbalanced by hypokalemia and hypochloremia—may result in a net conductivity that appears ostensibly stable. Consequently, the findings of CTI-ALPS in this study might reflect a state of compensated ionic homeostasis or a cancelation effect of heterogeneous ionic fluctuations, whereas DTI-ALPS captures the distinct mechanical failure of fluid transport.

### 4.2 ROI optimization and reproducibility

We investigated the reproducibility of DTI-ALPS and CTI-ALPS indices. We scanned DTI and MREPT in two young participants twice within a 30-min interval after moving the patient table. Both ALPS indices were calculated in three different ways, depending on different ROI sizes and different template applications. Firstly, the ALPS index was calculated by defining ROIs with a diameter of 3 mm in the implanting ROIs via subject-space. Secondly, the ALPS index was calculated by defining ROIs with a diameter of 5 mm in the subject imaging space. Finally, the ALPS index was calculated by defining ROIs with a diameter of 5 mm in the MNI space. A critical finding from our in-house repeatability test was the high sensitivity of ALPS metrics to ROI definition and spatial correspondence (Supplementary Table S5). We observed that fluctuations in ALPS values were strongly dependent on ROI size and the choice of brain template. The small inconsistencies in an ROI localization can substantially perturb the ALPS value.

Consistent with recent cross-vendor validations, our results suggest that using a 5 mm ROI diameter within the MNI template space provides a more stable and reasonable ALPS index than smaller ROIs or individual subject-space definitions. This ROI standardization is particularly relevant for DTI-ALPS, because small localization differences can disproportionately increase variance in directional diffusivities and weaken between-group inference. The higher measurement variability observed in CTI-ALPS likely contributed to its lower statistical power in group comparisons. However, this evaluation was performed in only two participants. Therefore, future studies should perform a test–retest validation in a larger sample, ideally across scanners/vendors, and adopt harmonized template-space ROI logic and standardized registration/QA procedures to improve measurement stability (Liu et al., 2024).

In general, the ALPS marker is confounded by multiple anatomical and microstructural factors (Schilling et al., 2025; Taoka et al., 2024). First, the radial asymmetry in white matter diffusivity is heavily influenced by underlying axonal geometry, including crossing fibers, anisotropic axonal dispersion, and axonal undulations, which can systematically inflate or alter ALPS indices independent of perivascular diffusion. Second, the ALPS-index inherently reflects predominant Brownian motion of water molecules in the radial direction, meaning that the reduced DTI-ALPS values observed in the AD group may partially reflect underlying structural white matter degeneration (such as axonal loss or demyelination) rather than exclusively representing perivascular fluid stagnation. While CTI-ALPS aims to provide a different physiological perspective based on ionic mobility, both indices are ultimately influenced by the complex microstructural deterioration inherent to Alzheimer’s disease. Therefore, it is important to interpret the ALPS index with caution, as it is an indirect proxy for perivascular fluid dynamics rather than a direct measurement of actual glymphatic clearance.

There are some limitations to translating the known limitations of DTI-ALPS to CTI-ALPS calculation. Because CTI-ALPS shares the identical mathematical framework and anatomical definitions as DTI-ALPS, the known methodological limitations of DTI-ALPS—specifically its high sensitivity to periventricular geometry, ROI definition, and registration accuracy—directly translate to CTI-ALPS. To account for this shared vulnerability, we utilized the standardized modified ALPS (mALPS) method using 5-mm ROIs in the MNI template space for both indices to minimize operator bias and ensure spatial consistency. Furthermore, these translated limitations are amplified by the lower spatial resolution of MREPT. Although both DTI and MREPT had the same in-plane resolution of 2 mm × 2 mm, our MREPT data were acquired with a 5 mm slice thickness, compared to the 2 mm slice thickness of DTI. This lower resolution exacerbates partial volume effects near the ventricles, inevitably blending the conductivity signals of adjacent structures (e.g., CSF) and increasing measurement variance. While we utilized a standardized MNI-space ROI approach to minimize operator bias, these geometrical vulnerabilities are inevitably amplified by the lower spatial resolution of MREPT. Consequently, this amplification of methodological noise provides a clear explanation for the lower statistical power of CTI-ALPS observed in our group comparisons.

### 4.3 Study limitations

This study has several limitations that should be considered when interpreting the findings and their generalizability. First, the study included 110 participants, which may limit the power to detect subtle differences in conductivity-based metrics. Second, despite using age as a covariate, the AD group was significantly older than the CN group. Such variance may be further accentuated in older cohorts, where aging-related conductivity changes could compress the effective dynamic range of CTI-ALPS and reduce sensitivity to disease-specific contrasts. Therefore, future studies should prioritize tighter age-matching to isolate disease-specific effects from healthy aging. Future studies with tighter age matching (and, where feasible, matching for other demographic factors) would provide cleaner group contrasts. Third, CTI-ALPS has not yet been verified using phantom or animal models, making it difficult to definitively interpret alterations in the context of neurodegeneration. Further work, including mechanistic validation with controlled ion manipulation in animal models, may help clarify the physiological determinants of conductivity as well as CTI-ALPS changes. Finally, the cross-sectional design limits our ability to determine if conductivity metrics can track disease progression or treatment response over time. Future research recommends a longitudinal study to determine whether conductivity-based metrics track treatment responsiveness or disease stability over time.

## 5 Conclusion

In this study, we evaluated conductivity-based glymphatic-related metrics derived from MREPT, focusing on CTI-ALPS, and compared them with diffusion-based DTI-ALPS across CN older adults, amnestic MCI, and mild-to-moderate AD. While DTI-ALPS remains the more sensitive marker for detecting perivascular water motion changes related to aging and cognition, CTI-ALPS offers a physiologically grounded extension of the ALPS framework. The trend toward lower CTI-ALPS in AD warrants further validation in larger, multi-site datasets with standardized registration and reproducibility-focused designs.

## Data Availability

The original contributions presented in the study are included in the article/Supplementary material, further inquiries can be directed to the corresponding author.

## Footnotes

1
https://dipy.org/
