# Supplementary material for: Evaluation of conductivity tensor image along the perivascular space in the brains of patients with cognitive impairments
Source: Front Aging Neurosci. 2026 Apr 28;18:1794175. doi: 10.3389/fnagi.2026.1794175 (PMC13162331; doi:10.3389/fnagi.2026.1794175)
Supplement: Supplementary file 1 [file Data_Sheet_1.docx]

**Evaluation of Conductivity Tensor Image (CTI) Along the Perivascular Space (ALPS) in the Brains of Patients with Cognitive Impairments**

**Supplementary Results**

**Supplementary Table S1. Summary of demographic characteristics and the neuropsychological test results of the participant groups**

| **Parameters** | **CN (1)** | **MCI (2)** | **AD (3)** | **Statistics** |
| --- | --- | --- | --- | --- |
| N | 30 | 52 | 28 | N/A |
| Age (years) | 70.6±5.3 | 72.1±6.2 | 76.8±9.2 | ***F=6.444, p=0.002 (1, 3) p=0.008***  ***(2, 3) p=0.026*** |
| Sex (Male/Female) | 13 (43.33%) /  17 (57.67%) | 13 (25.00%) /  39 (75.00%) | 6 (21.43%) /  22 (78.57%) | (1, 2) χ^2^=2.167, p=0.141  (1, 3) χ^2^=2.239, p=0.135  (2, 3) χ^2^=0.007, p=0.934 |
| MMSE(/30) | 28.37±1.45 | 27.06±1.50 | 19.00±4.63 | ***F=93.754, P<0.001 (1, 3) p<0.0001 (2, 3) p<0.0001*** |
| CDR | 0: 27 (90.00%) 0.5: 3 (10.00%) | 0.5: 52 (100%) | 0.5: 2 (7.14%) 1: 22 (78.57%) 2: 4 (14.29%) | N/A |

The continuous data are listed as mean ± standard deviation. *Italic* and **bold** characters show a statistically significant comparison.

Age was evaluated by ANOVA (F, p-value). MMSE scores were evaluated by ANCOVA (F, p-value) with age as a covariate. If any significant, then the post hoc test was performed using Bonferroni correction with p=0.05. Sex was tested by the chi-squared test.

The post-hoc results reflect the significant difference between CN and MCI groups (1:2), between CN and AD groups (1:3), and between MCI and AD groups (2:3).

*Abbreviations: cognitively normal (CN); amnestic mild cognitive impairment (MCI); Alzheimer’s disease (AD); Mini-Mental State Examination (MMSE); Clinical Dementia Rating (CDR)*

**
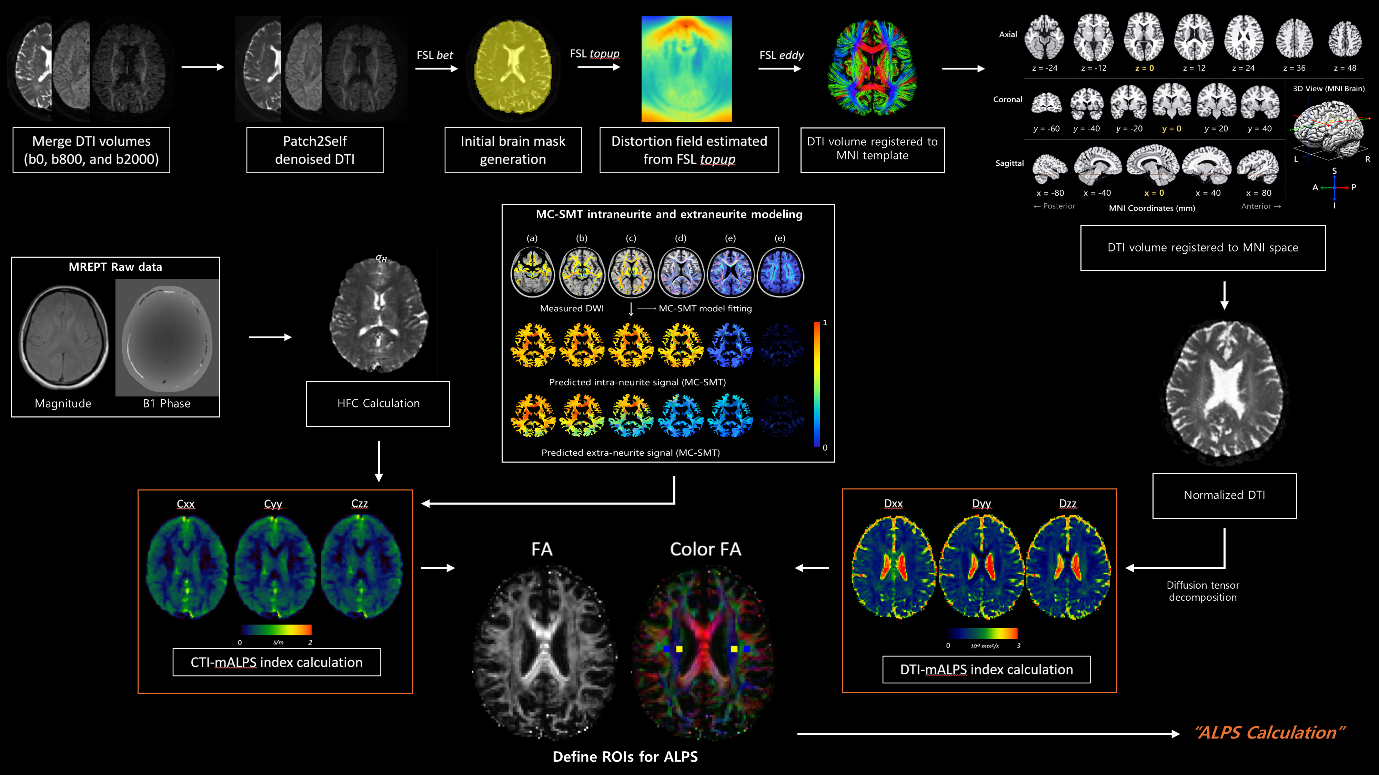
**

**Supplementary Figure S1. Flowchart of the image processing pipeline for CTI-ALPS and DTI-ALPS analyses.**

This flowchart summarises the end-to-end image processing workflow used in this study. It provides an overview of the major steps, including image acquisition, preprocessing/quality control, reconstruction of diffusion and conductivity-derived measures, definition of the ALPS-related ROIs, and extraction of the final CTI-ALPS and DTI-ALPS indices used for subsequent statistical analyses.

*Abbreviations: ALPS, analysis along the perivascular space; CTI, conductivity tensor imaging; DTI, diffusion tensor imaging; ROI, region of interest; QC, quality control; LT, left; RT, right; b-value, diffusion weighting factor;*


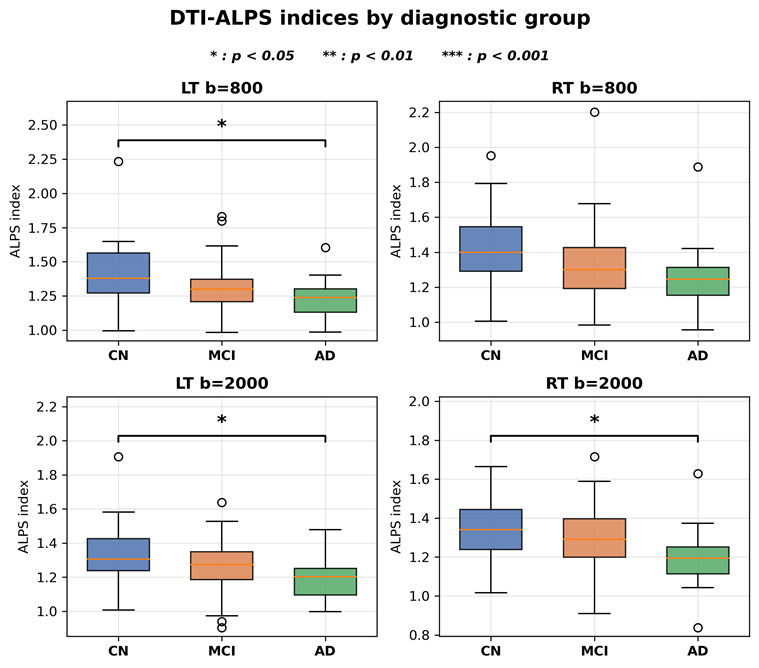


**Supplementary Figure S2. Group comparison results of DTI-ALPS measures between the three participant groups.**

*Abbreviations: Diffusion Tensor Imaging (DTI) along the perivascular space (ALPS); cognitively normal (CN); amnestic mild cognitive impairment (MCI); Alzheimer’s disease (AD); Left (LT); Right (RT);*

**Supplementary Table S2. Group comparison of laterality (Δ = RT−LT) of CTI-ALPS and DTI-ALPS indices.**

| **ALPS indices** | **b-value** | **CN (1)** | **MCI (2)** | **AD (3)** | **ANCOVA**  **by Group** |
| --- | --- | --- | --- | --- | --- |
| CTI-ALPS |  | -0.24 ± 0.12 | -0.18 ± 0.14 | -0.21 ± 0.16 | F=1.436, P=0.2424 |
| DTI-ALPS | b=800 | 0.02 ± 0.18 | 0.02 ± 0.14 | 0.01 ± 0.08 | F=0.009, P=0.9906 |
|  | b=2000 | 0.02 ± 0.14 | 0.03 ± 0.12 | 0.00 ± 0.07 | F=0.178, P=0.8372 |

The continuous variables are presented as mean ± standard deviation of Δ, where Δ is defined as the difference in ALPS index between right and left sides for each participant; Group differences in Δ were assessed using ANCOVA with age as a covariate. When applicable, post hoc pairwise comparisons were performed with Bonferroni correction (α=0.05).

*Abbreviations: Conductivity Tensor Imaging (CTI); Diffusion Tensor Imaging (DTI); analysis along the perivascular space (ALPS); cognitively normal (CN); mild cognitive impairment (MCI); Alzheimer’s disease (AD); Left (LT); Right (RT); difference (Δ).*

**Supplementary Table S3. Results of the correlation analysis between CTI-ALPS and DTI-ALPS measures.**

| ***Index*** | ***Side ROIs*** | **CTI-ALPS indices** | |
| --- | --- | --- | --- |
|  |  | **Left** | **Right** |
| ***DTI-ALPS Indices*** | | | |
| b=800 | Left | ***r=0.429, p≤0.001*** | ***r=0.412, p≤0.001*** |
|  | Right | ***r=0.413, p≤0.001*** | ***r=0.489, p≤0.001*** |
| b=2000 | Left | ***r=0.474, p≤0.001*** | ***r=0.485, p≤0.001*** |
|  | Right | ***r=0.424, p≤0.001*** | ***r=0.574, p≤0.001*** |

*Italic* and **bold** characters show a statistically significant comparison.

*Abbreviations: Diffusion Tensor Imaging (DTI) along the perivascular space (ALPS); Conductivity Tensor Imaging (CTI); Left (LT); Right (RT)*

**
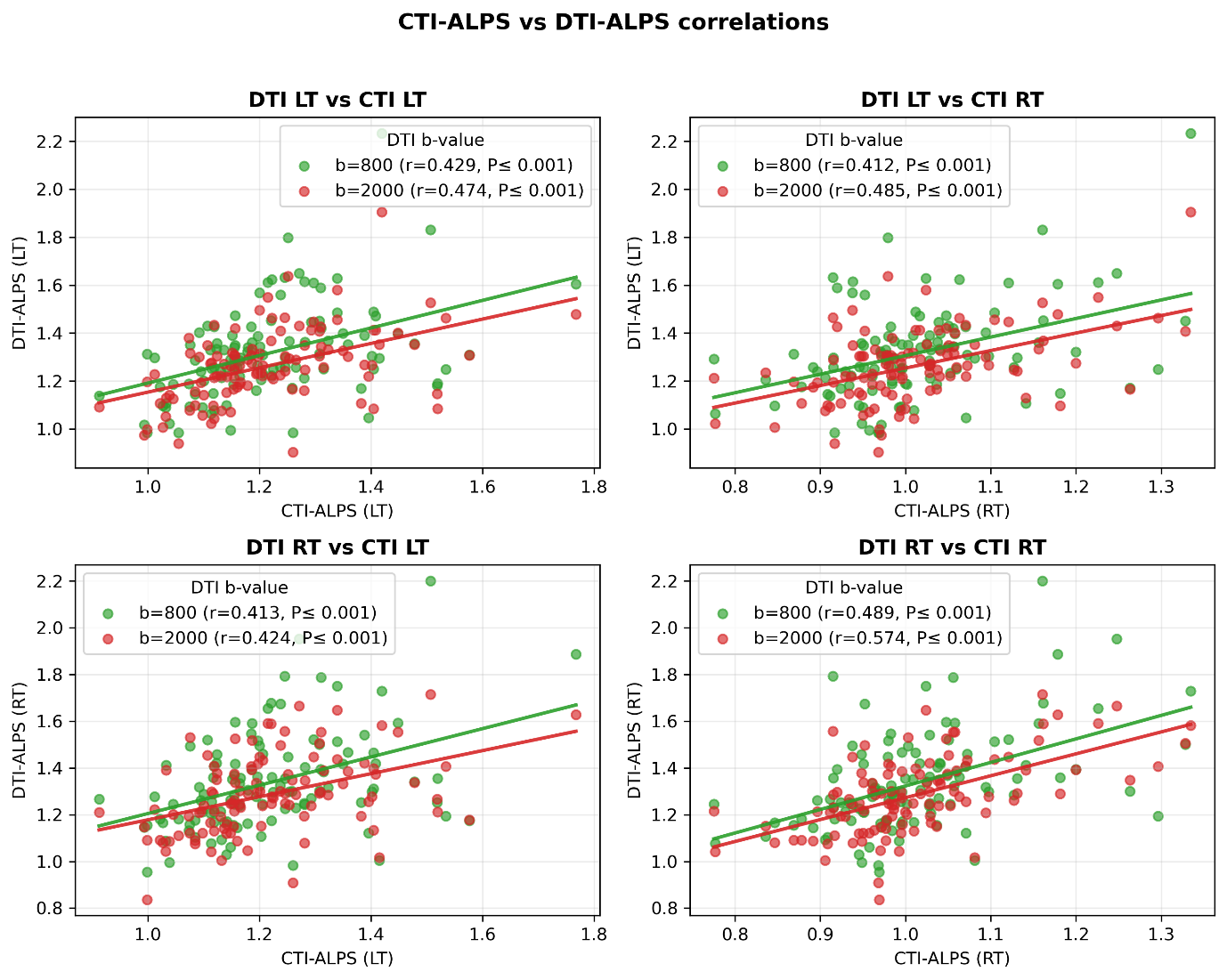
**

**Supplementary Figure S3. Results of the correlation analysis between CTI-ALPS and DTI-ALPS measures.**

Scatterplots show the relationships between CTI-ALPS and DTI-ALPS indices across hemispheres (LT/RT). For each panel, DTI-ALPS values at b=800 and b=2000 are plotted against the corresponding CTI-ALPS values, with least-squares regression lines overlaid. Pearson’s correlation coefficients (r) and associated p-values are reported in the legend for each b-value.

*Abbreviations: CTI, conductivity tensor imaging; DTI, diffusion tensor imaging; ALPS, analysis along the perivascular space; LT, left; RT, right; r, Pearson’s correlation coefficient; b, diffusion weighting factor.*

**Supplementary Table S4. Results of the Pearson correlation analysis** **between tensor components of CTI-ALPS and DTI-ALPS.**

|  |  |  | **CTI and DTI Tensor Compartments** | | |
| --- | --- | --- | --- | --- | --- |
| **Side** | **Fibers** | **DTI b-value** | ***Dxx-Cxx*** | ***Dyy-Cyy*** | ***Dzz-Czz*** |
| LT | Projection | b=800 | ***r=0.197, p=0.039*** | ***r=0.305, p=0.001*** | r=0.150, p=0.118 |
|  |  | b=2000 | ***r=0.370, p≤0.001*** | ***r=0.480, p≤0.001*** | ***r=0.406, p≤0.001*** |
|  | Association | b=800 | r=0.176, p=0.066 | r=0.012, p=0.897 | ***r=0.190, p=0.046*** |
|  |  | b=2000 | ***r=0.306, p=0.001*** | r=0.171, p=0.074 | ***r=0.287, p=0.002*** |
| RT | Projection | b=800 | ***r=0.383, p≤0.001*** | ***r=0.486, p≤0.001*** | ***r=0.421, p≤0.001*** |
|  |  | b=2000 | ***r=0.470, p≤0.001*** | ***r=0.588, p≤0.001*** | ***r=0.563, p≤0.001*** |
|  | Association | b=800 | r=0.181, p=0.058 | r=0.115, p=0.232 | ***r=0.243, p=0.011*** |
|  |  | b=2000 | ***r=0.298, p=0.002*** | ***r=0.371, p≤0.001*** | ***r=0.364, p≤0.001*** |

*Italic* and **bold** characters show a statistically significant comparison.

*Abbreviations: Diffusion Tensor Imaging (DTI) along the perivascular space (ALPS); Conductivity Tensor Imaging (CTI);*

**
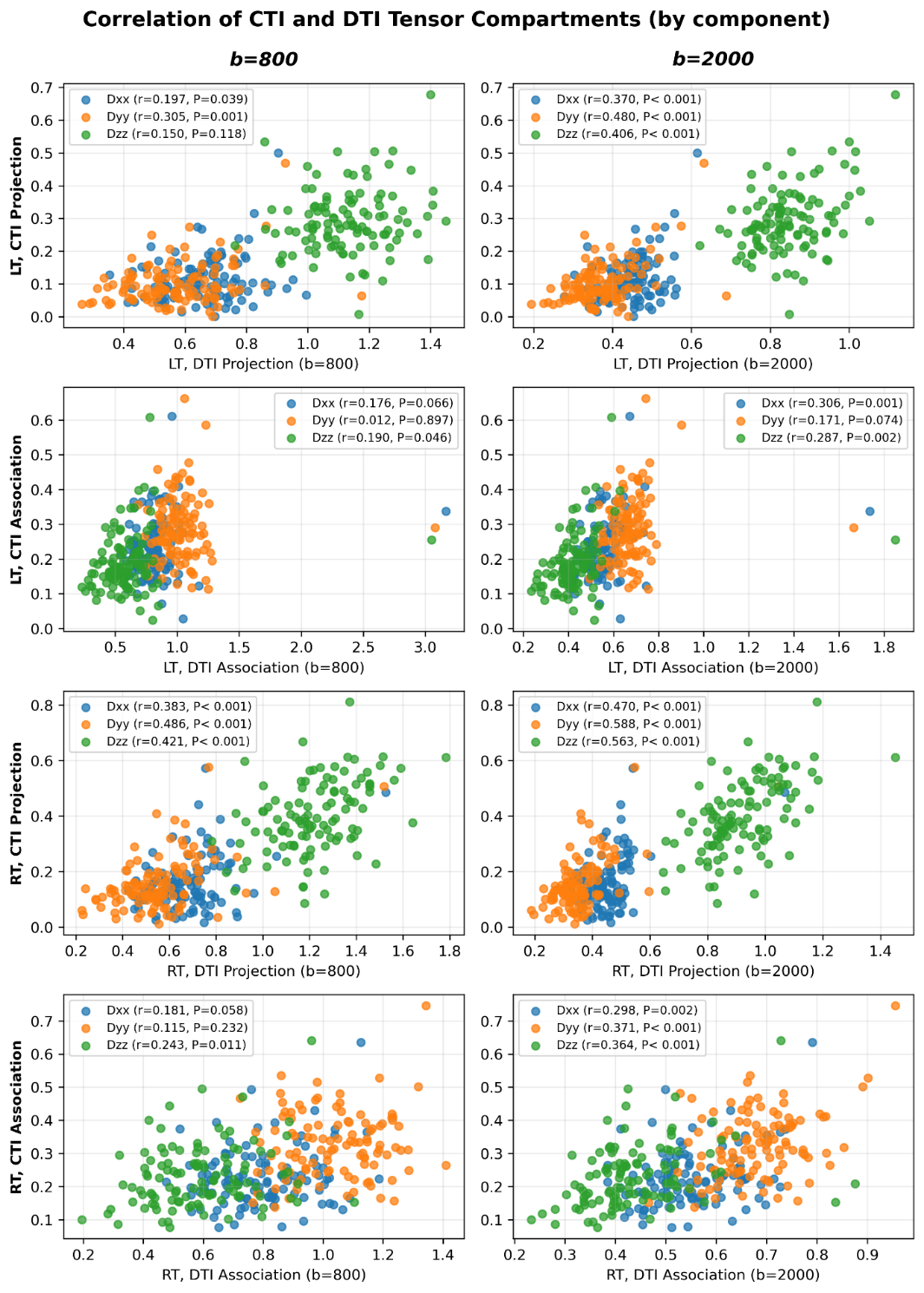
**

**Supplementary Figure S4. Results of the Pearson correlation analysis between tensor components of CTI-ALPS and DTI-ALPS.**

Scatterplots illustrate Pearson correlations between matched tensor components derived from CTI and DTI within projection and association fibre ROIs, separately for the left (LT) and right (RT) hemispheres. For each component pair (Dxx–Cxx, Dyy–Cyy, Dzz–Czz), relationships are shown at b=800 and b=2000, with fitted linear trend lines. Pearson’s correlation coefficients (r) and p-values are reported for each comparison.

*Abbreviations: CTI, conductivity tensor imaging; DTI, diffusion tensor imaging; ALPS, analysis along the perivascular space; LT, left; RT, right; ROI, region of interest; r, Pearson’s correlation coefficient; b, diffusion weighting factor; Dxx/Dyy/Dzz, DTI tensor components; Cxx/Cyy/Czz, CTI tensor components.*


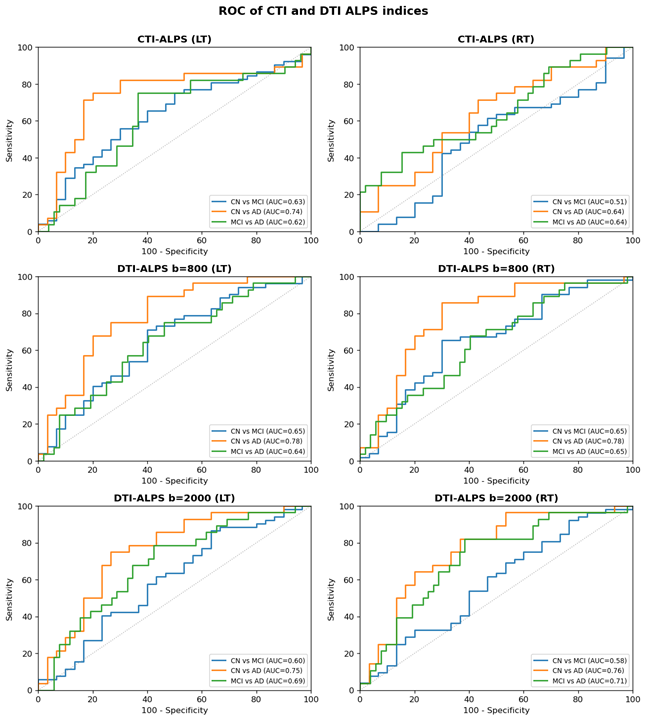


**Supplementary Figure S5. Graphical summary of the results of the ROC curve analysis of CTI-ALPS and DTI-ALPS.**

*Abbreviations: Diffusion Tensor Imaging (DTI) along the perivascular space (ALPS); Conductivity Tensor Imaging (CTI); cognitively normal (CN); amnestic mild cognitive impairment (MCI); Alzheimer’s disease (AD); Left (LT); Right (RT);*

**Supplementary Table S5. Variations of DTI-ALPS and CTI-ALPS indices between two measurements.**

| **Patient No.** | **ALPS indices** | **Side** | **b-value** | **3mm with imaging space** | **5mm with imaging space** | **5mm with MNI space** |
| --- | --- | --- | --- | --- | --- | --- |
| 1 | DTI-ALPS | Left | 800 | 313.81 %p | 17.69 %p | *10.30 %p* |
|  |  |  | 2000 | 10.87 %p | 16.21 %p | *14.28 %p* |
|  | CTI-ALPS |  | ALL | 20.73 %p | 30.06 %p | *23.68 %p* |
|  | DTI-ALPS | Right | 800 | 99.21 %p | 15.21 %p | *7.52 %p* |
|  |  |  | 2000 | 10.18 %p | 4.83 %p | *12.69 %p* |
|  | CTI-ALPS |  | ALL | 11.93 %p | 9.13 %p | *9.04 %p* |
| 2 | DTI-ALPS | Left | 800 | -18.67 %p | 0.11 %p | *-3.50 %p* |
|  |  |  | 2000 | -2.37 %p | -5.75 %p | *-7.42 %p* |
|  | CTI-ALPS |  | ALL | -1.83 %p | -7.29 %p | *-7.19 %p* |
|  | DTI-ALPS | Right | 800 | 4.35 %p | 10.04 %p | *3.52 %p* |
|  |  |  | 2000 | 5.58 %p | 3.29 %p | *-3.24 %p* |
|  | CTI-ALPS |  | ALL | -3.96 %p | -2.72 %p | *-1.99 %p* |

*Abbreviations: Diffusion Tensor Imaging (DTI) along the perivascular space (ALPS); Conductivity Tensor Imaging (CTI);*
